# Application of Grazing-Incidence X-ray Methods to Study Terrace-Stepped SiC Surface for Graphene Growth

**DOI:** 10.3390/ma15217669

**Published:** 2022-10-31

**Authors:** Boris S. Roschin, Tatiana S. Argunova, Sergey P. Lebedev, Victor E. Asadchikov, Alexander A. Lebedev, Yuri O. Volkov, Alexander D. Nuzhdin

**Affiliations:** 1 Federal Research Center “Crystallography and Photonics”, Russian Academy of Sciences, Leninsky ave. 59, 119333 Moscow, Russia; 2 Ioffe Institute, Russian Academy of Sciences, Polytekhnicheskaya st. 26, 194021 St. Petersburg, Russia

**Keywords:** graphene, epitaxial, SiC, terrace-stepped relief, X-ray scattering, atomic force microscopy

## Abstract

The synthesis of graphene by the graphitization of SiC surface has been driven by a need to develop a way to produce graphene in large quantities. With the increased use of thermal treatments of commercial SiC substrates, a comprehension of the surface restructuring due to the formation of a terrace-stepped nanorelief is becoming a pressing challenge. The aim of this paper is to evaluate the utility of X-ray reflectometry and grazing-incidence off-specular scattering for a non-destructive estimate of depth-graded and lateral inhomogeneities on SiC wafers annealed in a vacuum at a temperature of 1400–1500 °C. It is shown that the grazing-incidence X-ray method is a powerful tool for the assessment of statistical parameters, such as effective roughness height, average terrace period and dispersion. Moreover, these methods are advantageous to local probe techniques because a broad range of spatial frequencies allows for faster inspection of the whole surface area. We have found that power spectral density functions and in-depth density profiles manifest themselves differently between the probing directions along and across a terrace edge. Finally, the X-ray scattering data demonstrate quantitative agreement with the results of atomic force microscopy.

## 1. Introduction

A solid surface with an ordered nanorelief has been the subject of scientific and practical interest due to its potential use in nanoelectronic devices [1]. In addition, the nanorelief on a substrate surface affects the epitaxial growth. Sapphire (α-Al_2_O_3_) and silicon carbide (SiC) are examples of commonly used substrates whose polished vicinal surface is modified from a flat to a terrace-stepped structure by thermal or chemical treatment.

Techniques to enable probing surface properties typically use a probe located within a specific area of the sample. Atomic force microscopy (AFM) is indispensable for detecting the evolution of surface morphology. In particular, islet coarsening or terrace formation on sapphire substrates annealed in oxygen were studied in [2,3]. The authors of [4,5] illustrated the utility of AFM by analyzing nanosteps on a SiC surface annealed in hydrogen (H_2_) or hydrochloric (HCl) vapor. AFM provides reliable measurements in areas up to several tens of square microns. The need to go beyond a micrometer-scale mapping can only be satisfied if the sample moves sequentially in very small increments of motion, which is not practical. In contrast to AFM, grazing-incidence X-ray scattering (XRS) methods solve the problem of obtaining information about the entire surface of large samples.

The practical use of a nanostructured surface is moving forward rapidly in connection with the production of epitaxial graphene on SiC substrates (see, e.g., a review [6]). At a high temperature, the sublimation of Si atoms at a rate faster than C eventually results in the formation of epitaxial graphite film. A number of experiments have proved that the film is characterized by low mosaicity and good homogeneity [7,8,9,10]. However, some basic aspects involved in the graphitization process are still poorly understood.

The quality of graphene depends on the substrate preparation. Difficulties in understanding and predicting the evolution of morphology are, in particular, associated with a vicinal surface. High-vacuum annealing or thermal etching of SiC in H_2_ and HCl vapor leads to a terrace-stepped surface structure. The growth of graphene on a stepped surface of vicinal SiC can give reliefs that are more complex than terraces. The increased effectiveness of preparation procedures has raised the standard of graphene technology [11,12,13,14]. For instance, graphene layers with improved morphology were obtained under an argon atmosphere [9,15].

A common method of evaluation of roughness height, terrace period and thickness is AFM [4,5,11,12,13,14,15]. Unlike AFM, which is insensitive to a buried interface (for example, between the graphene layer and the substrate), XRS methods provide information at a depth of up to tens of nanometers. This depth value is at least an order of magnitude greater than AFM can give. XRS is preferable to AFM for the evaluation of stochastically distributed roughness [16]. In addition, the authors of [17,18,19] used highly sensitive XRS methods to study sapphire substrates with a terrace-stepped nanorelief. However, processed SiC surface is still a subject of investigation with this approach. Furthermore, there is still a lack of understanding of the interplay between values obtained from AFM and XRS data.

Probing a larger surface area is advantageous because it allows for a full set of parameters per sample, which results in improved characterization. In particular, one can estimate the period of terraces over the measurement area, the period dispersion, irregularities in terrace widths and material density variation across the depth. In this paper, we test the feasibility of using XRS under grazing incidence geometry and X-ray specular reflectometry to determine the surface structure and roughness parameters resulting from the vacuum annealing of SiC substrates. We compare effective roughness height, average terrace periods and deviations calculated according to XRS and AFM data and analyze the observed trends.

## 2. Materials and Methods

### 2.1. Sample Preparation

SiC (0001) wafers were purchased from different commercial channels. Semi-insulating crystals had a charge-unbalanced donor concentration of ~10^15^–10^16^ cm^−3^. Epipolished substrates with low miscut angles (≤2° off-axis) were certified as flat within commercial standards. After chemical-mechanical polishing a damaged layer remains on the surface of the substrate. To improve this situation we used ultra-high-vacuum (UHV) annealing in a closed tantalum cell. Before an experiment, a 4-inch diameter wafer was cut in equal samples 1 cm^2^ in area. The samples were cleaned and washed in distilled water and organic solvents.

Based on previous work in this area, we established the optimal temperature range that enables etching without changing the stoichiometric composition of the substrate surface. Namely, well-defined terraces emerge on a vicinal SiC surface at 1200 °C, when step-bunching is avoided, or it may be observed with higher offcut angles. A severe degradation takes place at 1800 °C [20,21]. Between surface restructuring and degradation, there are the most favorable conditions for obtaining atomically smooth terraces [13,22,23]. The annealing was carried out in the temperature range 1300–1500 °C at a residual pressure of 10^−4^ Pa.

A Si polar face of 4H-SiC was used to prepare the substrate. The growth of graphene on the Si face is well studied. Early experiments performed by grazing incidence X-ray diffraction [7], STM, LEED and spectroscopy [24,25] revealed the precursor phase for graphene growth on 6H- and 4H-SiC. Later, a schematized representation of the layer-by-layer graphene growth via the solid-state decomposition of the precursor [7] was improved. A new idea of the buffer layer between the graphene and Si-terminated surface of 4H-SiC resulted from X-ray reflectivity measurements [26]. The fact that the graphene on the Si-face is partly isolated from interactions with the substrate means that the sheet may grow perfectly continuously all around the surface. Meanwhile, the bi-layer graphene can replace the buffer layer in favor of better electronic properties [27]. Despite two decades of research, there are still issues to be clarified in relation to advanced processes or traditional treatments of a substrate surface.

### 2.2. Experimental Methods and Techniques

The AFM experiments were carried out using a P47 scanning probe microscope (NT-MDT Spectrum Instruments, Moscow, Russia). The operation of a hard probe was performed in the tapping mode. In addition, we used an independent method for determining the step heights in terrace-stepped nanostructures: high-resolution transmission electron microscopy (HRTEM). A Tecnai Osiris electron microscope (FEI Technologies Inc., Hillsboro, OR, USA) with 200 kV accelerating voltage was employed to investigate the cross-section samples. To prepare the samples we used a focused Ga^+^ ion beam provided by the FIB station. Before etching, a platinum film was deposited onto the surface of SiC.

The X-ray measurements were performed on a laboratory diffractometer. The setup was equipped with a mobile X-ray source-detector system in a “butterfly” geometry [28]. A conventional X-ray tube with a wide-aperture Cu-anode served as a source. Probing radiation was tuned to the wavelength of *λ* = 0.154 nm by the use of a Si(111) single-reflection monochromator providing Δ*λ*/*λ*~10^−5^. The experimental configuration included a three-slit collimator with vacuum paths to reduce the radiation absorption and scattering by air molecules. The collimator delivered the beam of width *d* ≈ 0.55 mm and divergence Δ*θ* ≈ 10^−4^ rad at total radiation intensity of *I* ≈ 3 × 10^6^ s^−1^.

The sample was placed on an adjustment table so that its surface was parallel to the propagation direction of the incident beam. During further measurements, the sample remained stationary while the source and detector rotated around the sample at specified angles. The angular position error of the source and detector rotation stages was kept within 2 arcsec. For data collection, a scintillation detector SCSD-4 (Radicon Ltd., St. Petersburg, Russia) was used in conjunction with a position-sensitive strip Dectris MYTHEN 2R (Dectris AG, Baden-Dättwil, Switzerland). It allowed us to measure the intensity of both X-ray beams reflected and scattered by the sample surface. We note that the scintillation detector provided better signal-to-noise ratio. It is important for measuring super-smooth surfaces since the scattering intensity declines within 8 orders of magnitude. The overall angle scanning range was 0–3°, while the angle of incidence for scattering experiments was set to 0.236°. The corresponding scattering vectors *q*_z_ and spatial frequencies *ν* covered a range of *q*_z_ = 0–0.42 Å^−1^ and *ν* = 0.05–10 μm^−1^, respectively.

To analyze the surface structure and roughness parameters with X-rays, we applied methods of specular reflectometry and grazing-incidence off-specular scattering. These methods are widely used for studying thin films and buried interfaces due to their angstrom-scale precision and high sensitivity to depth-graded and planar density distributions. Reflectivity and scattering data were acquired along each sample using the same setup.

**Figure 1:**
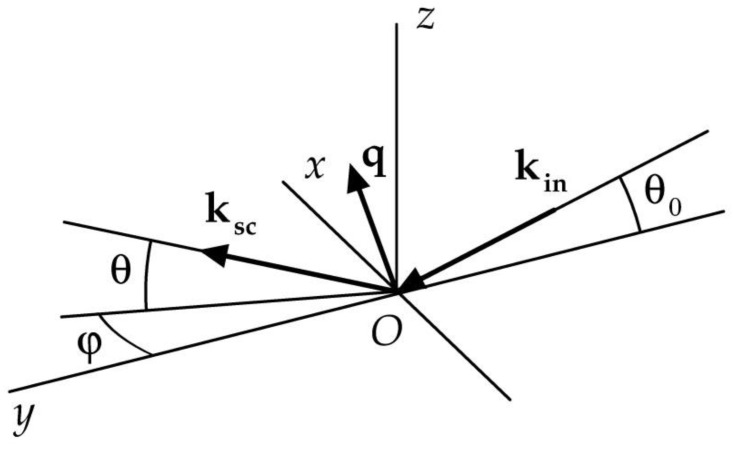
Schema of the X-ray scattering process and associated coordinate system. The origin point *O* is at the center of the illuminated region, the *x,y* plane coincides with the sample surface, the *z* axis is directed along the normal to the surface plane, and the *x* axis is perpendicular to the incident beam direction. The incident beam vector is **k**_in_; the grazing angle is *θ*_0_; the scattered beam vector is **k**_sc_; the scattering vector equals **q** = **k**_in_–**k**_sc_.

To represent the kinematics of X-ray scattering from a macroscopically flat horizontal surface under grazing incidence, we used the following coordinate system (Figure 1). The origin point *O* is the center of illuminated area. The *x,y* plane coincides with the air-sample interface. The *z*-axis is normal to the sample surface. The distribution of scattered intensity *Φ*(*θ*, *φ*) of a beam falling on the surface at a grazing angle of *θ*_0_ is expressed in terms of scattering amplitude *A*(***q***) by the following:

(1) $$\Phi \left(\theta ,\varphi \right)=\frac{1}{Q_{inc}}\cdot \frac{dQ_{sc}}{d\Omega }=\frac{{\langle |A\left(q\right)|}^{2}\rangle }{sin\theta_{0}\int d^{2}p}$$

where *Q_inc_* is the incident radiation power, *dQ_sc_* is the fraction of power scattered by the rough surface within the solid angle *dΩ*, ***q*** = *k*{cos*θ*cos*φ*;cos*θ*sin*φ*} is the projection of scattering wave vector onto the *x,y* plane, *k* = (2π/λ) is the wave vector, and ***p*** = (*x,y*) is the lateral coordinate vector. In turn, we assumed the spatial distribution of roughness along the surface to be independent from the density distribution normal to the surface, resulting in polarizability χ(***r***) = χ_0_Θ[z–ζ(***p***)], where ζ is the function describing the surface relief and Θ is the Heaviside step function. In that case, the exact expression for the scattering amplitude can be presented as [29]:

(2) $$A\left(q\right)=\frac{k^{2}}{2\pi }\int exp\left(-iqp\right)\Delta \chi \left(r\right)\psi_{0}\left(z,q\right)\Psi \left(r\right)d^{3}r$$

where *ψ*_0_ (*z*,*q*) is the incident wave, and *Ψ*(**r**) is the exact solution of the scattered wave equation. Expanding the polarizability parameter Δ*χ* into the series by small delta-like perturbations, the scattering amplitude in the first order can be written as [30]:

(3) $$A_{PT}\left(q\right)=\frac{k^{2}}{4\pi }\chi_{b}t\left(q_{0}\right)t\left(q\right)\int exp\left[i\left(q_{0}-q\right)p\right]\zeta \left(p\right)d^{2}p$$

where $\chi_{b}$ is the bulk polarizability within the sample, and *t*(*q*) is the amplitude transmission factor for the perfectly smooth interface, which can be calculated using, e.g., widely known Fresnel formalism.

It should be noted that at small grazing incident angles (*θ*_0_ << 1) the distribution of scattered intensity is significantly higher along the grazing angle *θ* than along the azimuthal angle *φ*. Therefore, it is practical to use the one-dimensional scattering diagram integrated over the azimuth, *Π*(*θ*) = ∫*Φ*(*θ,φ*)d*φ*. As shown in [30,31], the respective scattering distribution is presented as:

(4) $$\Pi \left(\theta \right)=\frac{k^{3}\chi_{b}^{2}}{16\pi sin\theta_{0}\sqrt{cos\theta_{0}cos\theta }}{\left|t\left(\theta_{0}\right)t\left(\theta \right)\right|}^{2}PSD\left(\nu \right)$$

Here *ν* = (2π)/|***q*** − ***q***_0_| is the spatial frequency along the surface plane, and PSD is the so-called power spectral density function of surface roughness, which in turn is the real part of the Fourier spectrum of autocorrelation function for height-to-height roughness [32]:

(5) PSD(ν)=4∫〈ζ(0)ζ(p)〉cos(2πνp)dp

As such, power spectral density for the surface roughness can be extracted directly from the diffuse scattering measurements. Note that for a quantitative comparison the effective roughness height *σ_eff_* can be defined by the relation:

(6) $$\sigma_{eff}=\sqrt{\int_{\nu_{min}}^{\nu_{max}}PSD\left(\nu \right)d\nu }$$

In turn, the angular distribution of specular reflectivity (for *θ* = *θ*_0_) from the surface within kinematical approximation is proportional to the depth-graded distribution of polarizability [33]:

(7) $$R\left(\theta \right)\equiv \frac{I\left(\theta \right)}{I_{0}}=R_{F}\times {\left|\frac{1}{\chi_{b}}\int_{-\infty }^{\infty }\frac{d\chi }{dz}exp\left(2iqz\right)dz\right|}^{2}$$

where *R_F_* is the classical Fresnel reflection from an ideally smooth step-like surface.

For the analysis of the experimental reflectivity data *R*(*θ*) and reconstruction of the electron density distribution ρ(*z*), we applied a model-independent approach proposed in [34], which is based on the extrapolation of the asymptotic component of reflectivity *R* into the range of large angles. One of the significant advantages of the said approach is that it does not require any prior assumptions on the sample internal structure in the form of a parametric model, but provides a direct numerical search of the depth-graded distribution of electron density instead. That makes model-independent methods useful for studies of disoriented and amorphous layers, where the choice of appropriate model is often ambiguous.

It should be noted that numerous recent works applied AFM data in conjunction with X-ray experiments to characterize interfaces, for example [35,36]. However, in these publications interfacial roughness has been only considered in terms of root-mean-square (rms) roughness height *σ*, which has several downsides. In particular, *σ* by definition is an integral parameter, and as such it does not account properly for neither stochasticity nor anisotropy of interfacial relief. Furthermore, estimations of *σ* from X-ray reflectivity data treat it as a fitting parameter in either Debye–Waller or Nevot–Croce factors, which *a priori* assume the interface to be fully stochastic, isotropic and having normally-distributed height-to-height roughness correlations. These assumptions, evidently, are not fulfilled for the oriented terrace-stepped relief. On the other hand, it has been previously shown [31,37] that the introduction of a non-Gaussian component into a height-to-height roughness distribution changes the integral specular reflection intensity in the asymptotic region up to one order of magnitude while preserving the same absolute *σ* value. As such, for non-stochastic non-isotropic interfaces estimation of *σ* from Debye–Waller or Nevot–Croce factors always contains a substantial error.

On the contrary, analysis of the interface roughness in terms of PSD function provides the spectrum of height-to-height correlations, which unambiguously describes the non-stochasticity of the roughness in question. In addition to that, the application of the grazing incidence XRS approach allows one to extract the PSD function directly from experimental data without any initial assumptions about its statistical properties. Furthermore, the extended self-consistent algorithm described in [38] allows one to account for the stochastic surface roughness during the calculation of ρ(*z*) by simultaneously processing both reflectivity and scattering data. All numerical X-ray calculations were implemented in the custom software written in Python language by the use of the Scientific Python and PyLab libraries and environment [39].

## 3. Results and Discussion

### 3.1. AFM and TEM

In this study we used the AFM/HRTEM complementary analysis to add more information to the previous statements concerning SiC surfaces slightly misoriented from on-axis. The authors of [40] established that the oxide was removed and minor scratches after mechanical polishing were partially healed during the first stage of annealing in vacuum at low temperatures of 900–1000 °C. From a temperature of 1200 °C terraces appeared between the scratches. Finally, flat terraces replaced the damaged layer on the surface of mechanically polished substrates. In the present paper, we investigated chemical-mechanical-polished (CMP) samples whose surfaces were similarly improved after vacuum annealing. Nevertheless, processing results showed clear differences between the smooth and scratched surfaces. Terrace propagation without interruption was observed when scratches were eliminated by CMP.

Attempts to control the terrace widths exhibit unsatisfactory aspects because miscut angles pertaining to different substrates vary slightly. At the same time, a manageable duration of annealing with an optimal fixed temperature allows for obtaining steps with a given height [22]. Annealing for 5 min. at a temperature of 1300 °C ends with the formation of steps with a height of *h* = 0.75 nm, which corresponds to the half unit cell of 6H-SiC. With the increase in the annealing time from 5 to 10 min. under the same temperature, the step height doubles and becomes equal to the unit cell: *h* = 1.5 nm. In addition, the controlled annealing experiments showed a gradual destruction of the terrace edges. Furthermore, the beginning of surface graphitization started above 1550–1600 °C.

The goal of current investigations was a direct measurement of step heights by HRTEM. The annealing of Si-face at 1450 °C led to the emergence of terraces and steps. The sample preparation for HRTEM analysis began with finding a low-index crystallographic direction, which is closest to the path of terrace propagation. A thin electron-transparent lamella was prepared by cutting a substrate along this direction. When the electron beam is parallel to a low-index zone axis, one obtains cross-sectional representations along six non-equivalent directions. Sequential alternation of steps was revealed under different viewing angles. However, the terrace width is not measurable in this configuration. The width may be less than or equal to that seen in the AFM maps.

**Figure 2:**
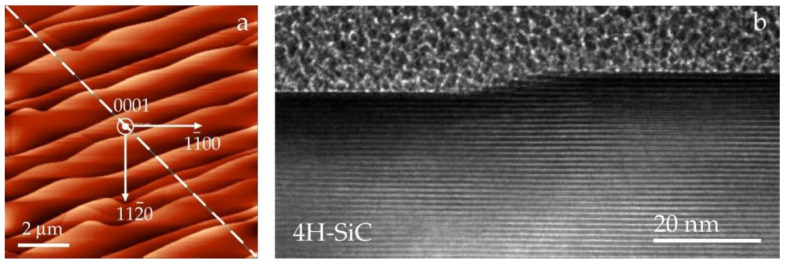
(**a**) AFM image of the terrace-stepped surface morphology of the Si-face of 4H-SiC. The annealing temperature is 1450 °C. The plot for height against distance (not shown) is obtained along the dashed line. (**b**) HRTEM image of a single step on a 4H-SiC surface imaged along a zone axis *[1-210]*. The period of the stripes corresponds to the unit cell parameter.

Figure 2a shows the AFM image of a typical step pattern in planar view. Well-defined terraces have kinks protruding in lateral directions. According to AFM data, the step heights may vary from 3 to 5 nm. The single step of a similar pattern in an HREM cross-section view is presented in Figure 2b. The value of the step height is 4 nm. This value corresponds to the step with a height of four layers of close-packed 4H-SiC. The layers themselves are seen between well-defined boundaries separated by a period of *c* = 1.005 nm. Throughout this substrate, other HRTEM estimates of the step heights are 3 and 5 nm.

Note that these steps have a rather large height. Graphene grown at the edges of high terraces and graphene between steps differ in their properties. A graphene device covering the surface step has a much higher resistance than the one on the terrace [41]. Mammadov et al. [42] and Lebedev et al. [43], using Kelvin-probe force microscopy, showed that the position of multilayer graphene, whose potential is higher compared to the monolayer graphene, corresponds to the borders of the terraces, while monolayer graphene covers the terraces.

The combination of minimum step height with maximum terrace width provides optimal conditions for obtaining homogeneous graphene layers [44]. Since these requirements are very tight and difficult to be met within the SiC industry, the problem arises of how to choose optimal thermal conditions for the growth of graphene. In particular, by controlling the heating rate, one can suppress the step-bunching process [45,46]. Lebedev et al. [43], using a heating rate of ~250 °C/min, obtained a homogeneous monolayer of graphene (with a minimum number of double-layer inclusions) on surface steps up to about several nanometers.

At increasing temperatures, the terrace-stepped relief may become more complex than at lower temperatures. The detection of changes requires probing a large area with nanoscale resolution. However, AFM or HRTEM provide information within a local probing range of a few tens of micrometers or a few nanometers, respectively. Meanwhile, the XRS setup described above makes it possible to inspect an area down to 10 × 10 mm^2^ in any location on samples 10 to 200 mm in size and obtain information about the entire surface.

### 3.2. Grazing-Incidence X-ray Scattering and Reflectivity Measurements

A commercial 4H-SiC substrate was compared with a UHV annealed (1550 °C) specimen cut out from the substrate. Scintillation and a position-sensitive strip detector measured the scattered intensity. Power spectral density (PSD) functions were extracted from the scattering data according to Equation (4). In addition, similar functions have been calculated directly from the AFM information according to Equation (5).

**Figure 3:**
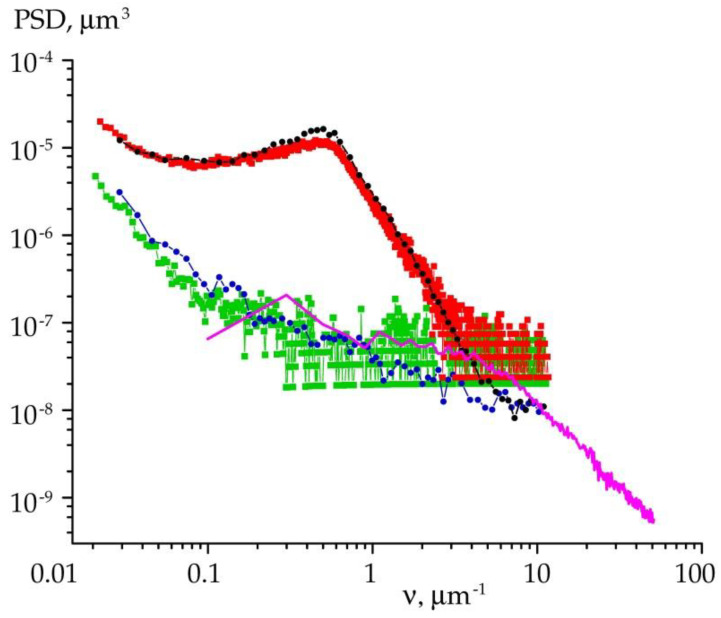
Roughness PSD functions from X-ray scattering measurements plotted against spatial frequency. Colored markers and lines represent the data obtained from the surface of polished (green, blue) and annealed (red, black) specimens with scintillation (circles) and position sensitive (squares) detectors. The solid magenta line defines PSD function calculated from AFM data for the polished surface.

As an illustration, in Figure 3 we show such PSD functions versus the spatial frequency. One can see that the scattering curves of the polished wafer, represented by the green and blue markers, decrease rapidly until they reach the background noise level. A fast descent of the PSD functions indicates that the wafer has isotropic roughness distribution over a well-polished atomically smooth surface. The effective roughness height calculated from Equation (6) equals *σ_eff_* = 1.6 Å. Very low roughness values result in the very small signal-to-noise ratio, which makes it hard to obtain reliable data with a strip detector.

Terrace-stepped surfaces were made by annealing samples as described above, following the procedures of Lebedev et al. [22,40]. The annealed surface may demonstrate the presence of different scales of roughness. Their physical justification relates to the appearance of large-scale roughness that is intrinsic to step formation, while low-scale part corresponds to the roughness of the terraces. In particular, the terrace formation made the roughness increase by almost an order in magnitude, yielding *σ_eff_* = 9.3 Å.

In Figure 3 the PSD functions of the stepped surface are drawn with red and black markers. In contrast to a polished face, the related curves have a convex form. The PSD functions exhibit characteristic maxima at *ν*_L_ ≈ 0.54 μm^−1^. The value *L* = 1/*ν*_L_ corresponds to the area-averaged period of the terrace-stepped nanostructure. Structural factors, such as the deviation of the terrace period from the average value and uneven terrace edges, broaden these maxima. We evaluated the deviation parameter Δ*ν*_L_ taking into account the full width at half maximum (FWHM) of the peaks, by fitting them with a standard Gaussian function. Since the AFM scan area is approximately three orders of magnitude smaller than the X-ray irradiation area, it turns out that the peak found from the AFM data is narrower. Because of that, the terrace period deviation is lower. Table 1 presents the resulting parameters.

**Figure 4:**
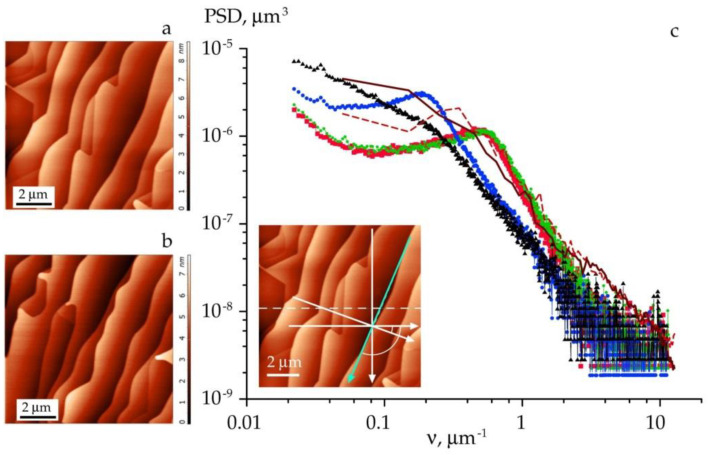
AFM images (**a**,**b**) and roughness PSD functions vs. spatial frequency (**c**) calculated for the surface of the annealed specimen (1450 °C). In the inset figure within (**c**) the dotted line indicates the initial AFM scan. The PSD functions along probing directions, which deviate 0°, −20°, −90° and −110° from the initial scan line, are represented with red, green, blue and black markers, respectively. Arcs mark angles of −20° and −110°. The cyan arrow shows the probing direction along a terrace edge. Brown lines depict the PSD functions extracted from AFM data. In particular, the solid brown line corresponds to the cyan arrow.

Terrace-stepped surfaces are highly anisotropic. X-ray scattering curves registered parallel to a terrace propagation direction may have features different to those seen along the orthogonal direction. To account for the anisotropic roughness characteristics of annealed specimens, we followed the methodology of Asadchikov et al. [19]. The scattering curves were measured under different lateral directions across the sample surface. The respective PSD distributions are presented in Figure 4. AFM images (Figure 4a,b) show the surface topography with well-defined terraces and kinks. The topography is not exactly the same for these two areas of the sample. Nevertheless, the kinks take place exclusively at the edges of steps and overall the terrace surface is essentially flat.

Processing of the scattering data showed that the integral value of the root mean square roughness remained in the range *σ_eff_* = 9.0 ± 0.6 Å. At the same time, the angular position of the peak was shifted according to azimuth rotation of the sample. Figure 4 illustrates the peak motion along the *ν*-axis. The shift is determined by a characteristic period *L* between the steps. From the peak position we obtained *L* = 1/*ν*_L_ ≈ 1.85 μm. In this case, the terraces extend along the direction marked in the cyan line in Figure 4, while the period is parallel to the orthogonal direction. Finally, we emphasize that the PSD peak is broadened along the *ν* axis more than 10 times, which indicates the presence of microscopic irregularities in the terrace width and shape across the surface of the sample.

The directions of AFM scans may exert a similar influence as above. These directions had different lateral angles with respect to the edges of the steps. From the scans, we calculated one-dimensional PSD functions. We noticed that the particular PSD functions extracted from the scan along a step edge have no peak. The corresponding distribution drawn with a solid brown line in Figure 4 is smoothed out and no peak is observed. The related rms roughness equals *σ*_eff_ = 8.3 Å. Meanwhile, the dashed brown line in Figure 4 exhibits a regular peak that corresponds to the area-averaged period of the terraces.

As we see from the examples above, grazing-incidence off-specular scattering allows one to reconstruct the statistical properties of lateral inhomogeneities, in particular of surface roughness and morphology. In turn, specular reflectometry offers the possibility of studying depth-wise inhomogeneities. By measuring the angular dependence of the reflection coefficient at small glancing angles one can obtain the depth-dependence of density *ρ* with a resolution of less than a nanometer.

**Figure 5:**
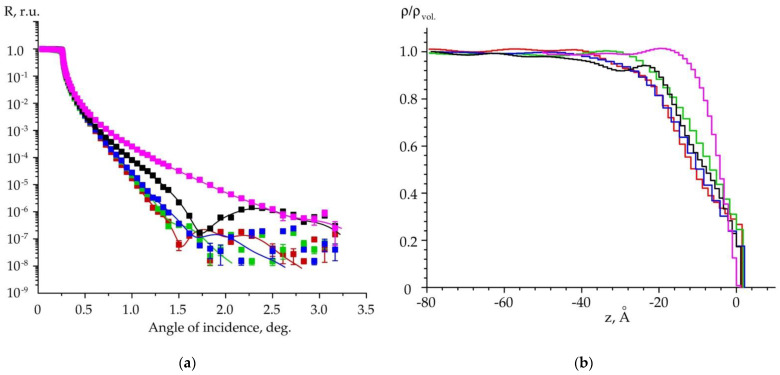
(**a**) Reflectivity curves measured for two samples: original substrate and annealed specimen (1450 °C). The magenta color defines the original substrate. The red, green, blue and black markers represent experimental data along the probing directions, which deviate 0°, −20°, −90° and −110°, respectively, from the AFM scan line in inset of Figure 4. The lines indicate fit results. (**b**) Distributions of depth-graded volumetric density, normalized to the density of bulk SiC, versus depth *z*.

In our present experiments, multiple reflectivity curves were measured along the same lateral directions that we used for the case of scattering. The results are shown in Figure 5a. Coloration represents a sample itself and the direction on the sample surface. In particular, magenta corresponds to the flat SiC substrate not subjected to annealing. Other colors indicate the relevant directions on the stepped surface of the annealed specimen. For example, we used black to show the intensity acquired along the step edges, while green markers represent an orthogonal direction. The caption of Figure 5 explains the variety of colors. Note that the asymptotic decay in intensity obtained for the annealed specimen fundamentally differs from that of the original substrate, which directly indicates the difference in morphology.

Depth-graded distributions of volumetric density are displayed in Figure 5b. Information was acquired from reflectivity data according to [34,38]. We found that the original sample has a disturbed surface layer as deep as 25 Å. The increased layer density may be due to the hardening effect caused by the surface-finishing treatment. In turn, density profiles for the annealed specimen reveal the presence of a so-called ‘transition layer.’ The layer depths vary from 40 to 60 Å, depending on the probing direction. We determined a pronounced change in density at a depth of 30 Å in the direction normal to the step edges. This value agrees with an average terrace height obtained from AFM scans.

There are, therefore, strong reasons to assume that redistribution of the probing beam intensity between specular (reflected) and off-specular (scattered) components depends on the lateral orientation of the sample. These components are provided by different parts of the terrace-stepped surface areas due to their inclination relative to the beam axis. This, in turn, corresponds to different ‘effective density’ values for a near-surface region averaged across the irradiated area. It should be noted that the more precise analysis of subsurface structure is constrained by non-uniform and irregular terrace edges. Various publications have studied and discussed the formation of a more regular surface relief under different treatments [4,14,40,47].

## 4. Conclusions

A detailed comparative analysis has shown that there is a good agreement between data obtained by AFM and grazing-incidence XRS methods. Meanwhile, the X-ray methods allow determination of not only lateral but also depth grade distribution of inhomogeneities. In particular, the oxidized layer with 25 Å thickness and the ‘transition layer’ were revealed. These near-surface regions are allegedly tied to the molecular adsorption from an ambient air medium. Furthermore, the depth-graded distributions of volumetric density provide an assumption that the oxidized layer manifests itself differently between the probing directions along and across a terrace edge.

X-ray scattering data is always averaged over an irradiation area significantly larger than the area measured in a regular AFM experiment. Due to this fact, we successfully extracted a number of meaningful statistical parameters, including effective roughness height, average terrace period and dispersion. Finally, a general structure of the oxidized and damaged near-surface layers was set out. Such information cannot be obtained by using conventional AFM or TEM methods.

An important aspect of X-ray measurements is the broad range of spatial frequencies associated with surface roughness. We stress that a detailed comparison of AFM and X-ray data would be possible if AFM measurements were conducted on numerous areas of different sizes with different linear scales. This, in turn, is challenging and takes longer than a regular X-ray scattering experiment.

## Figures and Tables

**Table 1:** Area-averaged period of terrace-stepped relief.

Average Terrace Period	Period Deviation, AFM	Period Deviation, XRS
1.85 μm	2.13 μm	4.2 μm

## Data Availability

Data is contained within the article.
